# Nox2-deficient Tregs improve heart transplant outcomes via their increased graft recruitment and enhanced potency

**DOI:** 10.1172/jci.insight.149301

**Published:** 2021-09-22

**Authors:** Silvia C. Trevelin, Anna Zampetaki, Greta Sawyer, Aleksandar Ivetic, Alison C. Brewer, Lesley Ann Smyth, Federica Marelli-Berg, Robert Köchl, Robert I. Lechler, Ajay M. Shah, Giovanna Lombardi

**Affiliations:** 1 King’s College London British Heart Foundation Centre, School of Cardiovascular Medicine and Sciences, London, United Kingdom.; 2 King’s College London, School of Immunology and Microbial Sciences, London, United Kingdom.; 3 University of East London, Health Sports Bioscience, London, United Kingdom.; 4 William Harvey Research Institute, Barts and The London School of Medicine and Dentistry, Queen Mary University London, London, United Kingdom.

**Keywords:** Cardiology, Immunology, Cell migration/adhesion, Heart failure, Immunotherapy

## Abstract

Nox2 is a ROS-generating enzyme, deficiency of which increases suppression by Tregs in vitro and in an in vivo model of cardiac remodeling. As Tregs have emerged as a candidate therapy in autoimmunity and transplantation, we hypothesized that Nox2 deficiency in Tregs in recipient mice may improve outcomes in a heart transplant model. We generated a potentially novel B6129 mouse model with Treg-targeted Nox2 deletion (Nox2^fl/fl^FoxP3Cre^+^ mice) and transplanted with hearts from CB6F1 donors. As compared with those of littermate controls, Nox2^fl/fl^FoxP3Cre^+^ mice had lower plasma levels of alloantibodies and troponin-I, reduced levels of IFN-γ in heart allograft homogenates, and diminished cardiomyocyte necrosis and allograft fibrosis. Single-cell analyses of allografts revealed higher absolute numbers of Tregs and lower CD8^+^ T cell infiltration in Nox2-deficient recipients compared with Nox2-replete mice. Mechanistically, in addition to a greater suppression of CD8^+^CD25^–^ T effector cell proliferation and IFN-γ production, Nox2-deficient Tregs expressed higher levels of CCR4 and CCR8, driving cell migration to allografts; this was associated with increased expression of miR-214-3p. These data indicate that Nox2 deletion in Tregs enhances their suppressive ability and migration to heart allografts. Therefore, Nox2 inhibition in Tregs may be a useful approach to improve their therapeutic efficacy.

## Graphical abstract

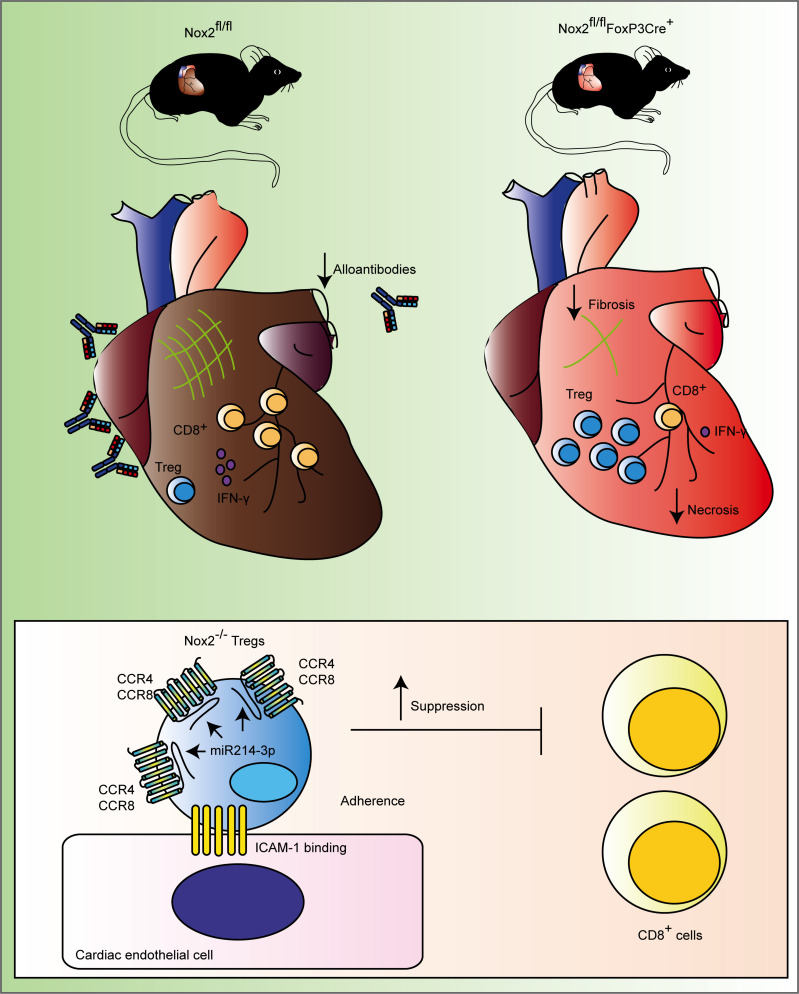

## Introduction

Cardiac transplantation remains the only available “curative” therapy for end-stage heart failure. However, the average survival after surgery is fewer than 10 years due to immune-mediated allograft rejection and side effects of immunosuppressive drugs (1). This provides the impetus to manipulate the immune system to achieve heart allograft tolerance (2).

Tregs are a subset of T cells expressing the transcription factor FoxP3 and the surface molecules CD4 and CD25 (3). In addition to maintaining self-tolerance (4), Tregs can recognize allogeneic MHC molecules and inhibit allograft rejection through different mechanisms (5). Tregs are currently under intensive investigation as an adoptive cell–based therapy to prevent transplant rejection and treat autoimmune diseases (6). Polyclonal Treg-based cell therapy approaches yielded promising early results for the prevention of graft-versus-host disease (7) and for curing type 1 diabetes (8, 9). We have also completed 2 phase I/II clinical trials, the ONE Study UK Treg Trial (NCT02129881) (10) and the Safety of Regulatory T Cell Therapy in Liver Transplant Patients (NCT02166177) (11), which assessed the safety and feasibility of adoptive transfer of ex vivo expanded polyclonal Tregs in renal and liver transplant patients (12–14).

Manipulation of Tregs in vitro can enhance their beneficial therapeutic effect (15, 16). Recently, our group showed that murine Nox2-deficient Tregs have higher suppressive activity in vitro on CD4^+^ T effector cell (Teff) proliferation than WT Tregs; this was attributed to increased nuclear levels of FoxP3 and NF-κB, which led to increased expression of CD25, CTLA-4, CD39, and CD73, key molecules linked to Treg suppressive function (17). An increased potency of Nox2-deficient Tregs was also manifest in vivo by reducing inflammation in a model of angiotensin II–induced (ANGII-induced) cardiovascular remodeling.

Having shown that Nox2 impairs Treg suppressive function (17), we hypothesized that targeting its deletion in FoxP3^+^ T cells of recipient mice could improve heart allograft survival.

## Results

### Generation of mice with Nox2 deletion in Tregs.

**Figure 1:**
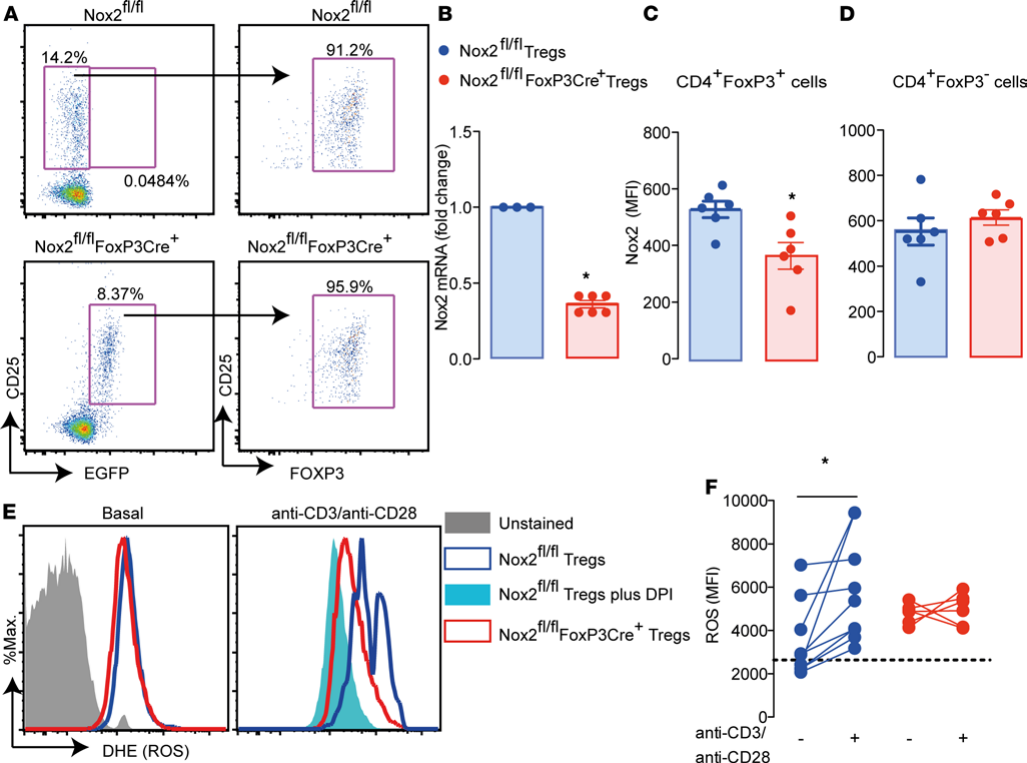
Generation of mice with Treg-targeted Nox2 deletion. (**A**) EGFP^+^ cells from lymph nodes of Nox2^fl/fl^FoxP3Cre^+^ mice stained with CD25 and FoxP3 antibodies. Plots are representative of 3 Nox2^fl/fl^FoxP3Cre^+^ and 3 Nox2^fl/fl^ mice. (**B**) Nox2 mRNA levels in lymph nodes (*n* = 3–6 per group). (**C** and **D**) Nox2 protein levels in CD4^+^FoxP3^+^ and CD4^+^FoxP3^–^ cells, respectively (*n* = 6 per group). (**E** and **F**) ROS estimated by dihydroethidium (DHE) fluorescence in purified Tregs stimulated with anti-CD3 (4 μg/ml) and anti-CD28 (8 μg/ml) antibody (*n* = 4–7 per group). Representative histograms are shown in **E**, and mean data is displayed in **F**. The dashed line in **F** represents the MFI of Nox2^fl/fl^ cells preincubated with the flavoprotein inhibitor diphenyleneiodonium (DPI; 10 μM) 30 minutes before stimuli. Data are shown as mean ± SEM. *P < 0.05 for indicated comparisons, Mann Whitney 2-tailed *t* test in **B**–**D** and Kruskal-Wallis followed by Dunn’s post test in **F**.

To analyze the contribution of Nox2 in Tregs to allograft protection, mice with FoxP3-targeted Nox2 deletion (Nox2^fl/fl^FoxP3Cre^+^ mice) were generated by crossing male B6129S-Tg(FoxP3eGFP/iCre)1aJbS/J mice (18) with female Nox2^fl/fl^ mice (19) (Supplemental Figure 1A; supplemental material available online with this article; https://doi.org/10.1172/jci.insight.149301DS1). Nox2^fl/fl^FoxP3Cre^+^ mice were confirmed to have Nox2 DNA recombination (Supplemental Figure 1B), and the EGFP^+^ cells in Nox2^fl/fl^FoxP3Cre^+^ mice were 95% CD25^+^FoxP3^+^ (Figure 1A). Nox2^fl/fl^FoxP3Cre^+^ mice also had lower Nox2 mRNA levels (Figure 1B) in lymph nodes and reduced Nox2 protein levels in CD4^+^FoxP3^+^ but not CD4^+^FoxP3^–^ cells (Figure 1, C and D). Purified Tregs from Nox2^fl/fl^FoxP3Cre^+^ mice did not increase ROS production after stimulation with anti-CD3ε and anti-CD28 antibodies, unlike cells from control Nox2^fl/fl^ mice. In fact, after stimulation, Tregs from Nox2^fl/fl^FoxP3Cre^+^ mice produced comparable ROS levels to Tregs from littermate controls treated with a Nox2 flavoprotein inhibitor, diphenyleneiodonium (Figure 1, E and F).

Under baseline conditions, Nox2^fl/fl^ control and Nox2**^fl/fl^FoxP3Cre^+^ mice had similar numbers of CD4^+^ and CD8^+^ cells in thymus, spleen, and mesenteric lymph nodes (Supplemental Figure 2, A–C), including naive (CD44^–^CD62L^+^ cells), effector memory (CD44^+^CD62L^–^ cells), central memory (CD44^+^CD62L^+^ cells), Th17 (CD4^+^RorγT^+^ cells), and CD4^+^ Tregs (CD25^+^FoxP3^+^ cells); CD8^+^ Tregs (CD8^+^FoxP3^+^ cells); and CD4^+^CD8^+s^ T cells (in thymus only). Nox2^fl/fl^FoxP3Cre^+^ and control Nox2^fl/fl^ mice had similar baseline heart and vascular function parameters (Supplemental Figure 2, D–F).

### Nox2 deficiency in Tregs improves allograft outcome.

**Figure 2:**
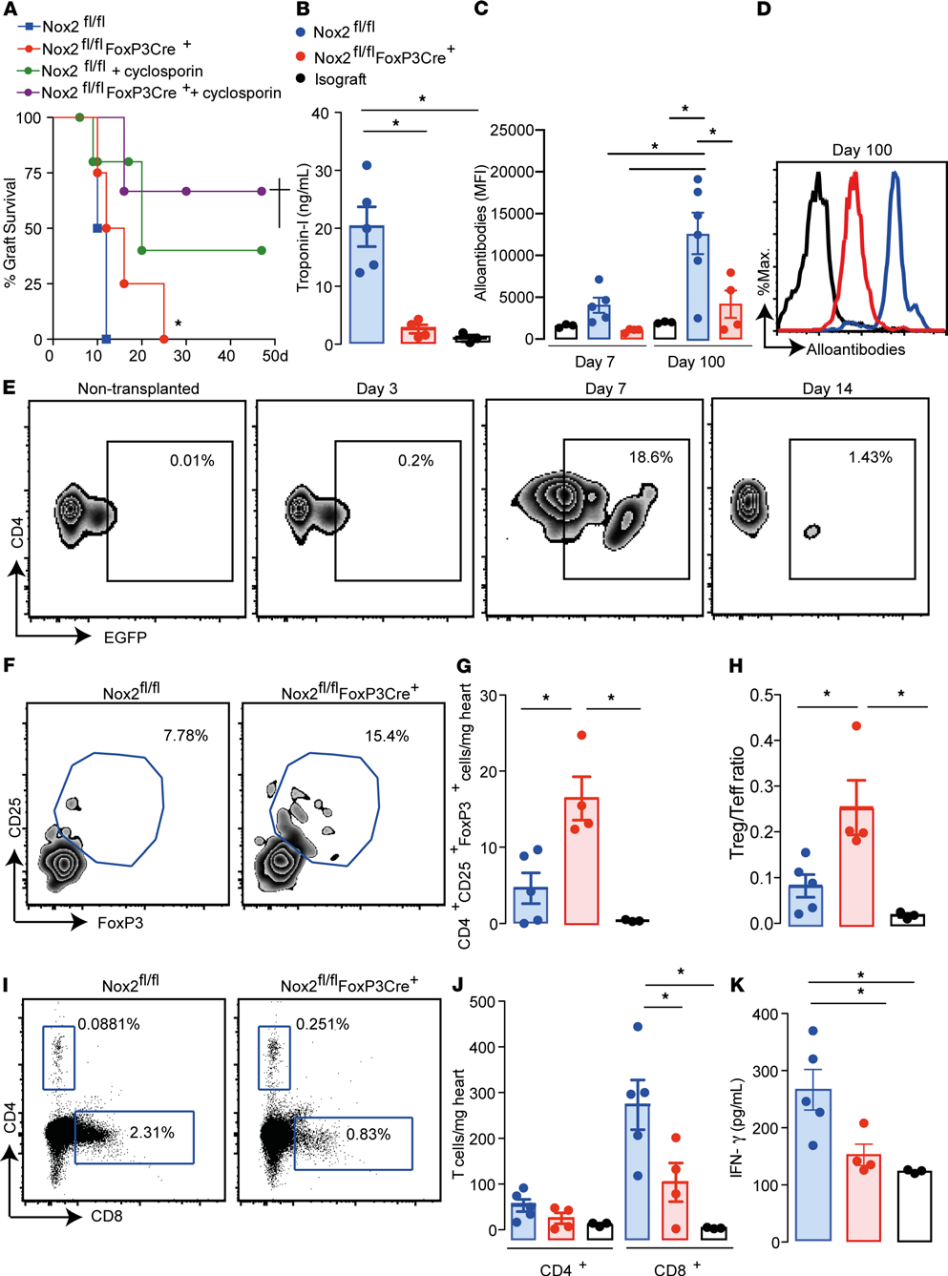
Heart allografts transplanted into Nox2^fl/fl^FoxP3Cre^+^ mice have higher Treg infiltration and better outcomes. Nox2^fl/fl^FoxP3Cre^+^ mice and littermate controls (Nox2^fl/fl^) were transplanted with hearts from CB6F1 mice. Mice transplanted with hearts from C57BL/6 mice were used as isograft controls. (**A**) Allograft survival curves. Some mice were treated daily with cyclosporin (30 mg/kg) s.c. for 10 days after transplantation (*n* = 4–5 per group). (**B**) Plasma troponin-I levels 7 days after transplant. (**C** and **D**) Plasma alloantibodies 7 and 100 days after the transplant. Representative histograms in **D** show the data obtained 100 days after transplant. (*n* = 3–5 per group). (**E**) Plots represent data from 3 allografts per time evaluated. The nontransplanted group represents data from 3 native hearts of naive nontransplanted mice. (**F** and **G**) Representative plots of CD25^+^FoxP3^+^ cells within the CD4^+^ cell population (**F**) and cells/mg of heart allograft 7 days after transplant (**G**). (**H**) Treg/Teff ratios. (**I** and **J**) Presence of CD4^+^ and CD8^+^ cells in transplanted hearts. Representative plots are shown in **I**, and cells/mg tissue is shown in **J**. (**K**) IFN-γ levels in heart allograft homogenates 7 days after surgery. Data are shown as mean ± SEM. †*P* < 0.05, *P < 0.05 compared with Nox2^fl/fl^ mice, Mantel-Cox test (**A**); *P < 0.05 for indicated comparisons in **B** and **C**, **G** and **H**, and **J** and **K**, using Kruskal-Wallis followed by Dunn’s post test (*n* = 3–5 per group).

Mice with FoxP3-targeted Nox2 deletion (Nox2^fl/fl^FoxP3Cre^+^ mice; H-2^b^) and littermate controls (Nox2^fl/fl^ mice; H-2^b^) were transplanted with hearts from CB6F1 mice (H-2^b/d^). Allografts transplanted into Nox2^fl/fl^FoxP3Cre^+^ mice showed delayed rejection as compared with those transplanted into littermate controls (Figure 2A and Supplemental Videos 1 and 2), along with diminished cardiomyocyte necrosis (Supplemental Figure 3A) and myocardial fibrosis (Supplemental Figure 3B) 7 and 100 days after surgery. To further confirm the relevance of Nox2 deletion in Tregs to the protection from heart allograft rejection, an animal model that more closely resembles the clinical setting was used. Recipient mice were treated with cyclosporin (30 mg/kg) for 10 days after heart transplantation. Both Nox2^fl/fl^ and Nox2^fl/fl^FoxP3Cre^+^ mice had increased allograft survival rates after cyclosporin treatment, but the Nox2^fl/fl^FoxP3Cre^+^ mice showed a lower rate of rejection (Figure 2A).

Plasma troponin-I levels were lower in Nox2^fl/fl^FoxP3Cre^+^ mice compared with littermate controls (Figure 2B) 7 days after transplantation. Plasma alloantibody levels were reduced in mice with Treg-targeted Nox2 deficiency 7 and 100 days after transplantation (Figure 2, C and D), while the inflammatory mediators, CCL2, IL-10, and IL-6, in allograft homogenates (Supplemental Figure 3, C–E) and CCL1 and CCL22 mRNA levels in heart tissues (Supplemental Figure 3, F and G) were equivalent between the 2 groups of mice 7 days after surgery.

To evaluate the contribution of Tregs to improved heart allograft outcome, the presence of FoxP3^+^EGFP^+^ cells was first analyzed in hearts transplanted into B6129S-Tg(FoxP3eGFP/iCre)1aJbS/J mice. The number of recipient FoxP3^+^EGFP^+^ Tregs in allografts started increasing 3 days after transplant, peaked at day 7, and then decreased by day 14 (Figure 2E). The increased number of Tregs at day 7 coincided with the higher FoxP3^+^ Treg numbers and Treg/Teff ratios observed in allografts transplanted in Nox2^fl/fl^FoxP3Cre^+^ mice compared with controls Nox2^fl/fl^ (Figure 2, F–H). The higher Treg number was associated with reduced CD8^+^ cells but not CD4^+^ T cells in the allografts (Figure 2, I and J). Moreover, Nox2^fl/fl^FoxP3Cre^+^ mice had lower IFN-γ levels in heart homogenates than Nox2^fl/fl^ mice 7 days after transplantation (Figure 2K). The numbers of CD4^+^, CD8^+^, and FoxP3^+^ Tregs in spleens were similar between Nox2^fl/fl^FoxP3Cre^+^ and Nox2^fl/fl^ mice (Supplemental Figure 4).

Therefore, Nox2 deficiency in Tregs improves heart transplant outcomes and prevents acute rejection through the reduction of CD8^+^ cell infiltration and IFN-γ production in the allografts associated with a higher proportion of Tregs.

### Nox2-deficient Tregs exhibit higher suppression of CD8^+^ T cell proliferation.

We have previously shown that Nox2-deficient Tregs inhibit in vitro CD4^+^ T Teff proliferation more efficiently than WT Tregs (17). To assess whether the reduced number of CD8^+^ T cells in the transplanted hearts in Nox2^fl/fl^FoxP3Cre^+^ recipient mice was due to a superior inhibitory function of Nox2-deficient Tregs, we purified and cocultured CD4^+^CD25^+^ Tregs with CD8^+^CD25^–^ Teffs. Tregs deficient in Nox2 inhibited CD8^+^ WT Teff proliferation (Supplemental Figure 5, A and B) more efficiently than WT Tregs (IC_50_ = 0.13 vs. IC_50_ = 0.41). Additionally, Nox-2–deficient Tregs abolished the production of IFN-γ by CD8^+^ Teffs, whereas a dose-dependent decrease was observed using different ratios of WT Tregs to WT Teffs (Supplemental Figure 5C). Therefore, Nox2^–/–^ Tregs exhibit higher suppression of CD8^+^ Teff proliferation and IFN-γ production than WT Tregs.

### Nox2 deficiency favors Treg migration and homing into heart allografts.

**Figure 3:**
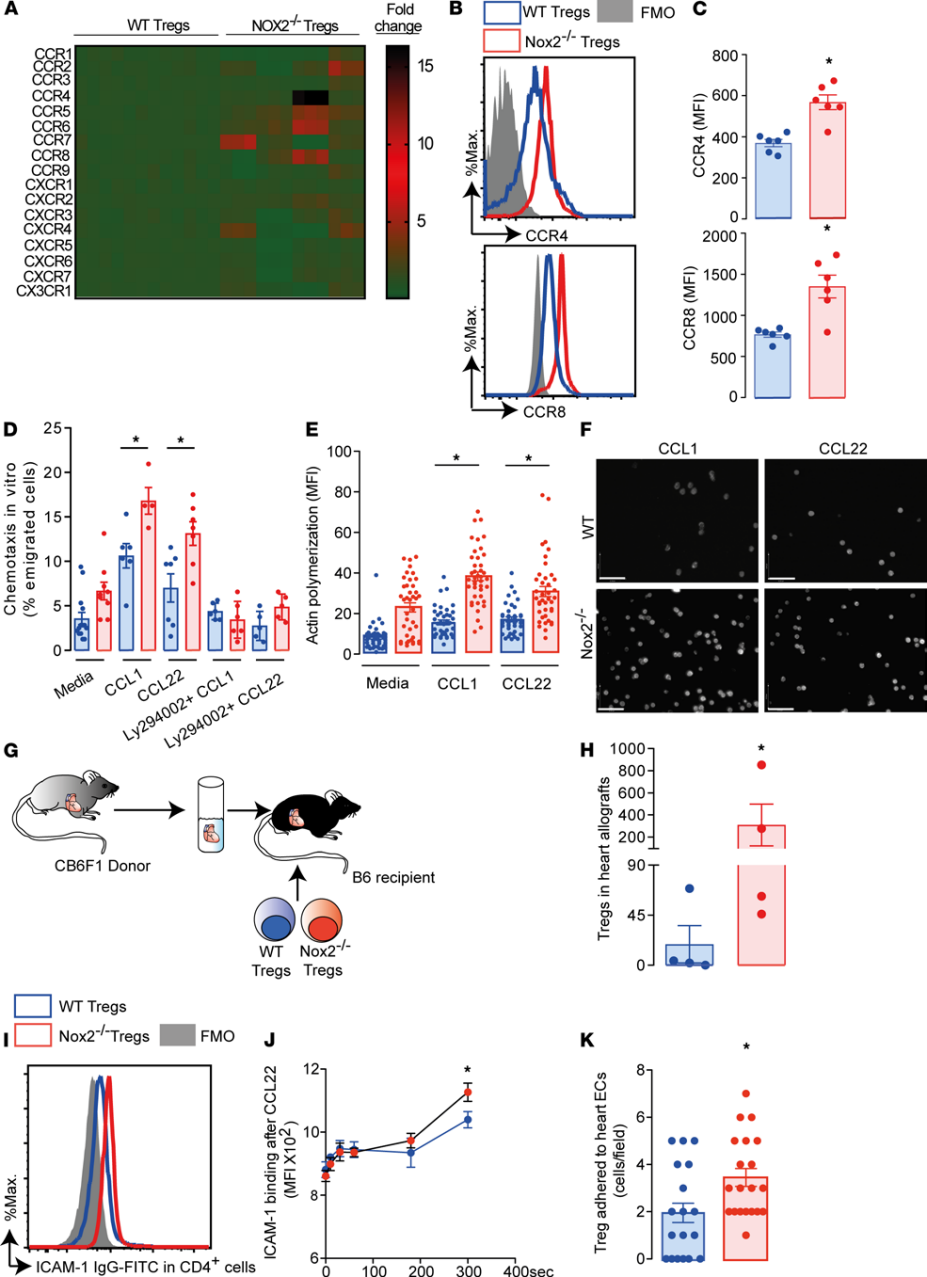
Nox2-deficient Tregs express higher levels of CCR4 and CCR8 than WT Tregs driving migration into heart allografts. Tregs were purified from spleens and lymph nodes of Nox2-deficient Tregs (Nox2^–/–^) and WT mice and assessed for (**A**) mRNA levels of chemokine receptors (*n* = 12); (**B** and **C**) CCR4 and CCR8 protein levels by flow cytometry (*n* = 6); (**D**) chemotaxis in vitro toward CCL1 and CCL22; and (**E** and **F**) actin polymerization stimulated by CCL1 and CCL22. Some cells were incubated with Ly294002 (5 μM). Full minus one (FMO) antibody was used as a negative control. Graphs and images represent 1 of 3 independent experiments. Scale bar: 32 μm. (**G**, **H**, and **K**) WT and Nox2^–/–^ Tregs were stained with different color cell tracers and tested for infiltration (after adoptive transfer) into CB6F1 hearts transplanted in C57BL/6 recipients (*n* = 4) (**G** and **H**) or adherence on cardiac ECs (**K**). (**I** and **J**) In vitro activation and binding of ICAM-1 in Tregs. Histograms and mean data represent 1 of 2 independent experiments. Data are shown as mean ± SEM. *P < 0.05 for indicated comparisons, Mann-Whitney 2-tailed *t* test in **C**, **H**, and **K**; Kruskal-Wallis followed by Dunn’s post test in **D** and **E**; and 2-way ANOVA followed by Bonferroni’s post test in **J**.

A potential mechanism underlying the increased number of recipient Tregs in allografts is augmented leukocyte trafficking. Chemokine receptors are essential for the initial phases of leukocyte trafficking (20) and were first analyzed in WT and Nox2-deficient Tregs purified from spleen and lymph nodes. These cells were predominantly thymus-derived Tregs, as approximately 70% of them were neurophilin-1^+^ (Supplemental Figure 6). Of the 17 chemokine receptors evaluated, 6 had enhanced mRNA levels in Nox2-deficient Tregs (Figure 3A). Among these, CCR4 is of particular interest, as it has been described as a homing receptor for the heart (21). The protein levels of CCR2, CCR4, CCR6, CCR7, CCR8, and CXCR4 were further investigated by multicolor flow cytometry (Figure 3, B and C, and Supplemental Figure 7, A and B). Nox2-deficient Tregs showed higher CCR4 and CCR8 expression than WT Tregs (Figure 3, B and C), along with a higher chemotactic index toward CCL22 and CCL1, respectively (Figure 3D). The difference in chemotaxis was abolished by preincubation of Tregs with the inhibitor of phosphoinositide 3-kinases (PI3Ks), Ly29002. PI3Ks are known downstream effectors of chemokine receptor signaling (22). Nox2-deficient Tregs also showed higher F-actin assembly following incubation with CCL1 and CCL22 (Figure 3, E and F). Of note, CCR2, CCR4, CCR7, CCR8, and CXCR4 protein levels in CD4^+^CD25^–^FoxP3^–^ and CD8^+^CD25^–^FoxP3^–^ Teffs were comparable between WT and Nox2-deficient mice (Supplemental Figure 7, C and D). To further confirm the superior migratory capacity of Nox2-deficient Tregs in vivo, WT and Nox2-deficient Tregs (H-2^b^), stained in green and orange, respectively, were adoptively cotransferred into C57BL/6 mice transplanted with CB6F1 hearts (Figure 3G). Supporting the previous results, a greater number of Nox2-deficient Tregs were recovered from the allografts as compared with WT Tregs (Figure 3H).

Chemokines mediate integrin activation via inside-out signaling and consequently induce adhesion of lymphocytes to ECs (23). Therefore, in addition to chemotaxis, an increase in numbers of Tregs in the allograft may also be influenced by their adherence to cardiac ECs. After stimulation with CCL22, Tregs deficient in Nox2 displayed higher binding in vitro to ICAM-1 than WT Tregs (Figure 3, I and J). Moreover, when WT and Nox2-deficient Tregs stained in contrasting color dyes were co-perfused over cardiac ECs, the Nox2-deficient cells displayed higher adherence to ECs (Figure 3K). Taken together, our data suggest that Nox2 expression in Tregs negatively regulates their chemotaxis and EC adherence.

### miR-214-3p drives increased CCR4 and CCR8 expression in Nox2-deficient Tregs.

**Figure 4:**
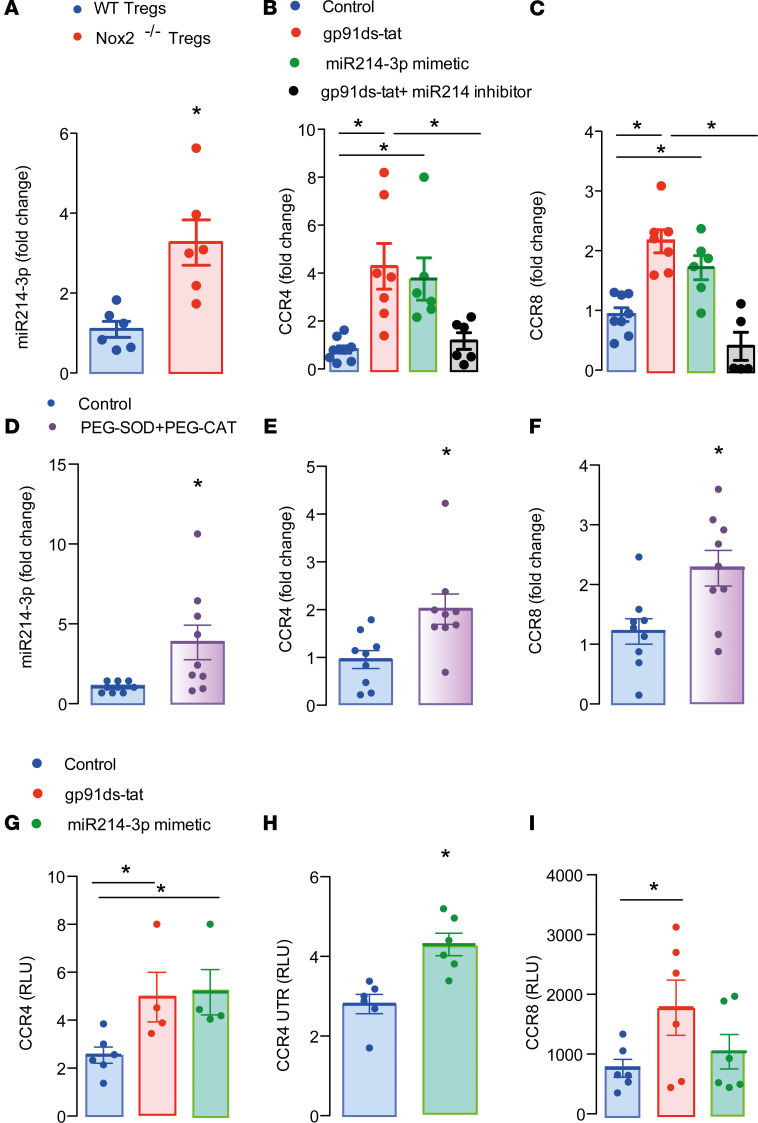
miR-214-3p is upregulated in Nox2^–/–^ Tregs and controls CCR4 and CCR8 mRNA expression. (**A**) Expression of miR-214-3p in Nox2^–/–^ or WT Tregs (*n* = 6). (**B** and **C**) CCR4 and CCR8 mRNA expression in Jurkat T cells transfected with miR-214-3p mimetic or inhibitor. Some cells were treated with the Nox2 inhibitor gp91ds-tat (30 μM) for 24 hours. Control cells were transfected with miR-negative control and incubated with sc-tat for 24 hours. Graphs represent 2 independent experiments. (**D–F**) Levels of miR-214-3p and mRNA coding for CCR4 and CCR8 in Jurkat T cells incubated 24 hours with PEG-SOD (20 IU/ml) and PEG-catalase (300 IU/ml). Graphs represent 3 independent experiments. (**G–I**) Reporter assay using CCR4, CCR4 untranslated region, and CCR8 constructs. Graphs represent 2 independent experiments. Data are shown as mean ± SEM. *P < 0.05 for indicated comparisons, Mann-Whitney 2-tailed *t* test in **A**, **D–F**, and **H** and Kruskal-Wallis followed by Dunn’s post test in **B**, **C**, **G**, and **I**.

The intracellular mechanism by which Nox2 controls transcription of CCR4 and CCR8 in T cells was next investigated. Because miRNAs are important regulators of transcription and a previous study showed that miR-214 deficiency decreases CCR4 expression in T cells (24), we assessed this as a possible mechanism. PCR analyses using primers for these miRs revealed that Nox2-deficient Tregs have higher expression of miR-214-3p but comparable levels of miR-214-5p to WT Tregs (Figure 4A and Supplemental Figure 8A). Accordingly, Jurkat T cells incubated with a specific Nox2 inhibitor, gp91ds-tat, had higher mRNA levels of CCR4 and CCR8 than those incubated with sc-tat peptide control. This increase was prevented in cells transfected with a miR-214-3p inhibitor (Figure 4, B and C). Transfection of Jurkat T cells with miR-214-3p mimetic also increased CCR4 and CCR8 mRNA levels (Figure 4, B and C), and cells treated with the Nox2 inhibitor showed higher miR 214-3p levels, which were reduced by transfection with the miR inhibitor (Supplemental Figure 8B). The incubation of Jurkat T cells with PEG-SOD and PEG-catalase also increased the levels of miR-214-3p as well as CCR4 and CCR8 mRNAs, indicating that the Nox2 effects were ROS dependent (Figure 4, D–F). We next cloned the 3′ untranslated region of mouse CCR4 and part of the coding region harboring binding sites for miR-214-3p in CCR4 and CCR8 mRNAs into a dual-luciferase reporter vector. Jurkat T cells transfected with the CCR4 constructs showed a higher luciferase signal in the presence of gp91ds-tat or the miR-214-3p mimetic (Figure 4, G and H), suggesting increased CCR4 mRNA stabilization. The assays using the CCR8 construct showed an increased signal in the presence of gp91ds-tat but not miR-214-3p mimetic, pointing to a possible distinct regulatory mechanism (Figure 4I).

**Figure 5:**
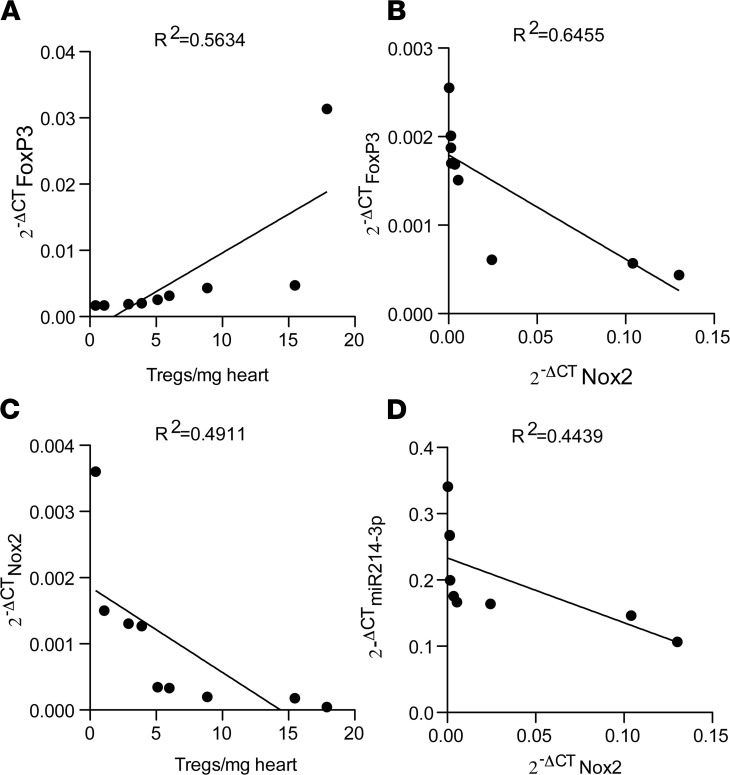
Nox2 expression inversely correlates with expression of FoxP3 and miR 214-3p. Linear correlation between (**A**) FoxP3 mRNA and Treg counts in heart allografts, (**B**) FoxP3 mRNA and Nox2 mRNA, (**C**) Nox2 mRNA and Treg counts in heart allografts, and (**D**) miR-214-3p expression and Nox2 mRNA (n = 9) 7 days after transplantation. Values of cycle threshold (2^–ΔCT^) for miR-214-3p were normalized by miR-let-103; Nox2 and FoxP3 were normalized by GAPDH. The *R*^2^ values are displayed on the top of each correlation.

Finally, we studied the expression of mRNA for Nox2, FoxP3, and miR-214-3p in heart allografts 7 days after transplantation. The expression of mRNA for FoxP3 in the allografts directly correlated with the Treg counts (Figure 5A) and inversely correlated with the expression of mRNA for Nox2 (Figure 5B). In agreement with the results presented in Figure 2, F and G, and Figure 4A, Nox2 mRNA expression inversely correlated with Treg counts and with miR-214-3p expression in the heart allografts (Figure 5, C and D).

Taken together, our results indicate that Nox2 deficiency in Tregs improves heart allograft outcomes due to a greater suppression of CD8^+^ Teff proliferation and IFN-γ production. Additionally, Nox2-deficient Tregs migrate more efficiently into the allografts due to their increased expression of CCR4 and CCR8 mRNAs mediated via miR-214-3p.

## Discussion

The development of improved methods to suppress allograft rejection is a major goal to enhance the effectiveness of the life-saving cardiac transplant therapy. Previous work, including early phase clinical studies, suggests that the administration of Tregs may be one way to induce immune tolerance and improve allograft outcome (7, 11). We focused on the ROS-generating enzyme Nox2, as we have recently found it to reduce Treg suppression of CD4^+^ Teff proliferation (17) and thereby increase cardiovascular inflammatory responses. Here, a potentially novel mouse model with Treg-specific deficiency of Nox2 in the recipient showed improved allograft outcomes, which were accompanied by reduced cardiomyocyte necrosis, lower myocardial fibrosis, and diminished circulating levels of alloantibodies. The mechanisms underlying these improved outcomes were increased chemotaxis and adherence of Tregs in the transplanted hearts as well as an enhanced suppression of CD8^+^CD25^–^ Teff proliferation by Nox2-deficient CD4^+^CD25^+^ cells. Additionally, Nox2-deficient Tregs downregulated IFN-γ production in cultures with CD8^+^ Teff cells, which could also have contributed to increase survival of the heart allografts (Supplemental Figure 9).

Nox2 was previously shown to be involved in leukocyte migration in distinct disease contexts and related to different cell types, including ECs (25), platelets (26), and neutrophils (27). In the present study, we observed that Nox2 deficiency upregulates CCR4 expression in CD4^+^CD25^+^ Tregs, which favors their infiltration into heart allografts. The importance of Treg chemotaxis toward CCR4 ligands in the context of heart allograft survival has been corroborated by previous studies (28–30). Long-term allograft survival induced by treatment with tanshinol plus rapamycin was reversed by neutralizing the CCR4 ligand CCL22 (29). Furthermore, Lee et al. (28) showed that upregulation of CCR4 and Treg infiltration of the transplant following combined anti-CD154 monoclonal antibody and donor-specific transfusion induced tolerance, which was not observed in CCR4-deficient recipients or in mice receiving anti-CD25 antibody treatment.

It is well known that CCR4 inside-out signaling activates the integrin CD11a, which adheres more to ICAM-1 expressed by antigen-presenting cells (APCs) and ECs (31, 32). In fact, we observed that Nox2-deficient Tregs had enhanced binding to ICAM-1 in vitro after CCL22 stimulation as compared with WT Tregs. As a consequence, Tregs deficient of Nox2 had a higher adherence to cardiac ECs, facilitating migration into allografts; the possible increased interaction with APCs, reducing their capacity to provide costimulatory signals, could have contributed to a higher suppression of CD8^+^ Teff proliferation. Corroborating the importance of integrin activation to heart allograft survival, Warren et al. (32) showed that anti-α4 integrin antibody reduced the number of Tregs in transplanted hearts, leading to impaired allograft survival. The increased CCR8 expression displayed by Nox2-deficient Tregs could also have contributed to enhanced chemotaxis to allografts and to the higher suppressive function. In support of this, Barsheshet et al. (33) showed that the suppressive function of CD25^+^CD127^lo^ Tregs in vitro is upregulated by expression of CCR8 and the presence of its ligand CCL1 (33). Nox2-deficient Tregs showed enhanced expression of mRNA for 6 different chemokine receptors, despite only CCR4 and CCR8 having corresponding increases in protein levels as compared with WT Tregs. These discrepancies could be due to internalization and degradation of chemokine receptors (22).

We further explored the intracellular mechanism through which Nox2 regulates CCR4 and CCR8 expression in T cells and found that miR-214-3p is enhanced in Nox2-deficient Tregs. Our data support an miR-dependent upregulation of target mRNA transcription or stabilization previously reported in the literature (34–36). Consistently, hearts from miR-214–deficient mice displayed lower CCR4 expression compared with WT controls (24), indicating upregulation rather than reduction of mRNA levels. Additionally, Nox2 mRNA levels in heart allografts inversely correlated with miR-214-3p levels, Treg counts, and FoxP3 mRNA expression. Our results agree with those of a previous study showing an association between decreased expression of miR-214-3p and increased levels of alloantibodies and development of bronchiolitis obliterans syndrome following lung transplantation (37). Additionally, murine heart allografts had lower levels of miR-214-3p compared with isografts (38).

The higher Treg infiltration in recipient mice with Treg-targeted Nox2 deletion was associated with lower necrosis and fibrosis of heart allografts and with lower plasma levels of troponin-I as early as 7 days after surgery. Indeed, increased troponin-I levels correlated positively in patients with acute heart transplant rejection (39), which was also observed in the murine heterotopic heart transplant model used in this study.

The lower interstitial fibrosis observed in allografts transplanted into recipient mice with Nox2 deletion agrees with our recent published data showing that the adoptive transfer of Nox2-deficient Tregs induced lower cardiac fibrosis in a model of ANGII-induced cardiovascular remodeling (17). It also agrees with findings from another study showing Treg depletion using anti-CD25 antibody aggravated cardiac fibrosis in a model of virus-induced myocarditis, whereas adoptive transfer of Tregs prevented it (40).

In addition to impaired T cell mediated alloresponses, there was a significant decrease in levels of alloantibodies in mice with Nox2 deficiency in Tregs as compared with controls with preserved Nox2 activity. Because posttransplantation-reactive anti-HLA antibodies in humans are associated with the development, frequency, and severity of cardiac allograft vasculopathy, it would be of interest to assess whether Nox2 deficiency in Tregs also affects this serious complication after heart transplantation (41).

We did not see differences in CD4^+^ T cell infiltration between allografts transplanted into Nox2^fl/fl^ and Nox2^fl/fl^FoxP3Cre^+^mice. This response differs from the pattern of T cell infiltration in a model of ANGII-induced cardiac remodeling, in which Nox2-deficient mice had a decrease in both CD4^+^ and CD8^+^ Teffs (17). These differences could be attributed to the distinct importance of CD4^+^ and CD8^+^ cells in different animal models. In fact, cytotoxic CD8^+^ T cell responses against mismatched MHC class I alloantigen are the principal arm of the cellular response against the transplanted organ (42), whereas cardiac remodeling is modulated mainly by CD4^+^ T cells producing IL-17 (43).

Nox2-deficient Tregs express higher levels of CCR4 and CCR8, but other chemokine receptors were not affected (CCR2, CCR7, CCR6, and CXCR4; Supplemental Figure 7, A and B). Therefore, the presence of CCL1 and CCL22 in the heart transplant microenvironment increases the potential therapeutic effects of Nox2-deficient Tregs in this context. Because Nox2 deletion does not reduce the expression of other chemokine receptors, other disease contexts where CCL1 and CCL22 have a secondary role probably would not be negatively affected by Nox2 deletion in Tregs.

In conclusion, we observed that Nox2 deficiency increased the suppressive capacity and chemotaxis of Tregs in vitro and in vivo (Supplemental Figure 9). Therefore, Nox2 could be used as a target to potentiate Tregs with clinical application.

## Methods

### Mice and in vivo studies.

Nox2^fl/fl^FoxP3Cre^+^ mice were generated by crossing B6129S-Tg (FoxP3-EGFP/iCre)1aJbs male mice with Nox2 homozygous floxed female mice (19). Heterotopic heart transplants were performed as previously described (44).

### ELISA.

Troponin-I (High sensitivity mouse cardiac Troponin-I ELISA kit, Life Diagnostics, CTNI-1-HSP) and IFN-γ (Mouse IFN-γ DuoSet ELISA, R&D Systems, DY485-05) levels were determined by ELISA according to manufacturer’s recommendations.

### Treg purification.

CD4^+^CD25^+^ cells were purified from spleens and lymph nodes using a commercial kit (Dynabeads FlowComp Mouse CD4^+^CD25^+^ Treg, catalog 11463D).

### Flow cytometry.

Levels of alloantibodies in plasma and single-cell analyses of heart allograft digests were determined in a LSRFORTESSA flow cytometer (BD Biosciences) and analyzed using FlowJo software 9.7.5. Superoxide production was estimated using 10 μM dihydroethidium (17).

### Quantitative PCR.

RNA was extracted using TRIzol Reagent (Thermo Fisher, 1559626). SYBR green real-time PCR was performed using the ΔΔCt method and GAPDH for normalization. cDNA synthesis and quantitative PCR for miR-214-3p and miR-214-5p were done using an miCury LNAtm MiRNA PCR starter kit, mmu-miR-214-3p, and mmu-miR-214-5p (Qiagen, product 339320, YKP-MM-YP00204510-YP00204575).

### Cell transfection.

Jurkat T cells were transfected with MicroRNA mimic miR-214-3p (hsa-miR-214-3, Thermo Fisher, MC12124) or hsa-miR-214-3p miRCURY LNA miRNA Inhibitor (hsa-miR-214-3p miRCURY LNA miRNA Inhibitor, Qiagen, YI04105004-ADA) or miRVanatm miR Mimic, Negative Control (Thermo Fisher, 4464058) by electroporation (Lonza, VPA-1002).

### Statistics.

Analyses were performed using GraphPad Prism software v9.0. Comparisons were undertaken using Kruskal-Wallis followed by Dunn’s post test or a Mann Whitney 2-tailed *t* test or 2-way ANOVA followed by Bonferroni post test, as appropriate. A Mantel-Cox test was used to compare survival rates. *P <* 0.05 was considered significant.

### Study approval.

All animal procedures were undertaken in accordance with the Guidance on the Operation of the Animals (Scientific Procedures) Act, 1986 (UK Home Office), and with institutional ethics approval from King’s College London.

See Supplemental Methods for additional information.

## Author contributions

AMS and GL supervised the study. AMS, GL, and SCT conceived the study and contributed to the experimental design. SCT, AZ, AI, ACB, LAS, RK, and GS performed experiments and interpreted the data. FMB and RIL provided critical intellectual input. SCT, GL, and AMS wrote the manuscript.

## Supplementary Material

Supplemental data

Supplemental video 1

Supplemental video 2

## Version 1 08/10/2021

In-Press Preview

## Version 2 09/22/2021

Electronic publication
