# Supplementary material for: Nox2-deficient Tregs improve heart transplant outcomes via their increased graft recruitment and enhanced potency
Source: JCI Insight. 2021 Sep 22;6(18):e149301. doi: 10.1172/jci.insight.149301 (PMC8492330; doi:10.1172/jci.insight.149301)
Supplement: Supplemental data [file jciinsight-6-149301-s165.pdf]

Nox2-deficient Tregs improve heart transplant outcome via their increased graft recruitment and enhanced potency

Silvia C. Trevelin[1,2], Anna Zampetaki[1], Greta Sawyer[1], Aleksandar Ivetic[1], Alison C. Brewer[1], Lesley Smyth[3], Federica Marelli Berg[4], Robert Köchl[2], Robert I. Lechler[2], Ajay M Shah[1*], Giovanna Lombardi[2*].

[1]King's College London British Heart Foundation Centre, School of Cardiovascular Medicine and Sciences, London, United Kingdom; [2]King's College London, School of Immunology and Microbial Sciences, London, United Kingdom; [3]University of East London, Heath Sports Bioscience; [4]William Harvey Research Institute, Barts and The London School of Medicine and Dentistry, Queen Mary University London, London, United Kingdom.

***Corresponding authors:** Professor Giovanna Lombardi, Immunoregulation laboratory, MRC Centre for Transplantation, 5th Floor Tower Wing, Guy's Hospital, London SE1 9RT, UK. Tel: 0207 1887674. Email: giovanna.lombardi@kcl.ac.uk; Professor Ajay M. Shah, School of Cardiovascular Medicine & Sciences, James Black Centre, 125 Coldharbour Lane, London SE5 9NU, UK. Tel: 0044-207848-5189; Fax: 0044-207848-5193. Email: ajay.shah@kcl.ac.uk.

Running title: Nox2-deficient Tregs improve heart allograft outcome

Material and methods

Mice and in vivo studies. $Nox2^{fl/fl}FoxP3Cre^{+}$ and littermate controls $Nox2^{fl/fl}$ ($H\text{-}2^{b}$) received hearts from CB6F1 mice ($H\text{-}2^{bd}$). Donors were acquired from Charles River and maintained in the local animal facility. Heterotopic heart transplants in abdominal cavity were performed in 8 to 10-week-old mice as previously described (1) and graft viability evaluated by palpation and echocardiography. $Nox2^{fl/fl}FoxP3Cre^{+}$ mice were generated by crossing B6129S-Tg (FoxP3-EGFP/iCre)1aJbs males acquired from Jackson laboratories (strain 023161) with Nox2 homozygous floxed females(2). Gene-modified mice were compared with matched $Nox2^{fl/fl}Cre^{-}$ ($Nox2^{fl/fl}$) littermates. B6129S-Tg (FoxP3-EGFP/iCre)1aJbs mice express EGFP and the codon optimized "humanized" Cre recombinase under the control of the mouse FoxP3 promoter in a bacterial artificial chromosome vector (3), therefore $Nox2^{fl/fl}FoxP3Cre^{+}$ mice had Treg-specific targeting without interference with the FoxP3 gene, which is located in chromosome X near to the Nox2 gene (4).

Echocardiography. Two-dimensional echocardiography was performed in isoflurane anaesthetized mice with an 8 to 12MHz ultrasound probe (Visualsonics Vevo 2100). Best imaging was obtained in the transplanted hearts by setting gain high and the compression low. Videos of long- and short- axis were acquired and recorded for further analyses of graft viability each 2 days up to 10 days after transplant and once a week thereafter.

Histology. Fibrosis and necrosis were analysed in paraffin-embedded sections stained with Picrosirius Red and ApopTag Red in situ apoptosis detection kit (Milipore; Cat. S7165), respectively. Sections for necrosis evaluation were also stained with anti- cardiac troponin T antibody clone 1C11 (Abcam; Cat. Ab8295), donkey anti-mouse IgG (H+L) conjugated to Alexa 488 (Invitrogen; Cat.R37114) and DAPI. Images were acquired on a DM200 LED bright field or confocal TCS SP5 (Leica, London UK) microscope. Image J software was used for analyses (version 1.0 for Mac OS X).

Detection of alloantibodies. Plasma from mice submitted to heart transplant was obtained by centrifuging the blood at 650g for 15min after collection in tubes with heparin (50IU/ml blood). The plasma was kept at minus 80° C until further analyses. Samples were diluted 1:80 and incubated with $2.5x10^5$ anti-CD3 PE (Cat.552127, BD BD Biosciences, Oxford, UK) pre-stained splenocytes from Balb/c mice for 20 minutes at 4 °C. The cells were washed in PBS and incubated for additional 20 minutes at 4 °C in the presence of anti-mouse IgG (Fc specific)-FITC antibody produced in goat (1:200, Sigma; Cat. F5897). The cells were then washed twice in PBS and acquired in a BD LSRFortessa™ flow cytometer (BD Biosciences, Oxford, UK). Plasma samples incubated with $2.5x10^5$ anti-CD3 PE pre-stained splenocytes from B6 mice were used as negative controls.

Flow cytometry. Hearts were perfused with phosphate buffered saline (PBS) through the left ventricle before harvesting. Single cell suspensions were prepared by digesting hearts in a mixture of collagenase (1mg/ml, Cat.C5138 , Sigma Aldrich, St. Louis, USA), DNase (160 IU/ml, Cat.DN25 , Sigma Aldrich, St. Louis, USA),) and hyaluronidase (500 IU/ml, Cat.H3884 , Sigma Aldrich, St. Louis, USA),) at 37° C for 30 minutes. Samples were homogenized and sequentially filtered through a 40 µm nylon mesh. Red blood cells were lysed in 2% NH_4Cl buffer. Non-specific interactions were blocked with anti-mouse CD16/CD32 Ab (10 µg/mL, 1:50, eBioscience, Waltham, USA, Cat. 14-0161-82) prior to staining. Anti-CD4 PercP clone RM4.5 (1:100, Cat. 561090), CD8 APC-Cy7 (1:100, Cat. 561967), CD45 FITC (1:100, Cat. 553080), Nox2 (Cat. 1:100, 611414), CD25 PE (1:50, Cat. 553075), CCR6 BV711 (1:100, Cat. 740646) antibodies were purchased from BD Biosciences (Wokingham, UK). Anti-FoxP3 APC (1:50, Cat.17-5773-82) antibody was acquired from eBioscience (Waltham, USA). Anti-CD45 BV510 (1:50, Cat. 103137), CCR2 BV785 (1:100, Cat.150621), CCR4 PE-Cy7 (1:100, 131213), CCR8 FITC (1:100, Cat.150313), CCR7 BV605 (1:100, Cat. 120125), CXCR4 BV421 (1:100, Cat. 146511), Neurophilin-1 BV421 (1:50, Cat. 145209) antibodies were acquired from Biolegend (San Diego, CA). Anti-mouse Alexa 633 (1:400, Cat. A21052) was

acquired from Invitrogen (Carlsbad, USA). FoxP3 transcription factor staining buffer set-kit (Cat. 00-5523-00) from eBioscience was used for intracellular staining.

Samples were acquired in a LSRFORTESSA flow cytometer (BD Biosciences, Oxford, UK) and analysed using FlowJo software 9.7.5 (Ashland, USA).

In vitro suppression assay. Teffs ($CD8^{+}CD25^{-}$) and Tregs ($CD4^{+}CD25^{+}$) were purified from spleen and lymph nodes using commercial kits (ThermoScientific, Dynabeads^{TM} Untouched^{TM} CD8 cells, Cat. 11417D; naïve Dynabeads^{TM} FlowComp^{TM} Mouse $CD4^{+}CD25^{+}$ Treg, Cat. 11463D). Antigen presenting cells (APCs) were obtained by incubation of total splenocytes with anti-CD4 and anti-CD8 antibodies (clones YTS191.1 and YTS169.4, respectively) followed by negative selection using dynabeads from Dynabeads^{TM} FlowComp^{TM} Mouse $CD4^{+}CD25^{+}$ Treg kit. $CD8^{+}CD25^{-}$ Teffs were stained with Violet cell tracer (Cell Trace^{TM} Violet Cell Proliferation Kit, Cat.C34557) and incubated with Tregs under stimulation by anti-CD3ε (4 µg/ml; BD Biosciences, Wokingham, UK, Cat. 553238) and APCs (1:2).

Cell transfection. Jurkat T cells (clone EC6-1, ATCC TIB-152) were stimulated with Dynabeads Human T activator CD3/CD28 (1:2) for 3 hours. Cells were then transfected with microRNA mimic miR-214-3p (hsa-miR-214-3; ThermoFisher Scientific, Cat. MC12124) or hsa-miR-214-3p miRCURY LNA miRNA Inhibitor (Qiagen, Cat. YI04105004-ADA) or miRVana^{tm} miR mimic, negative control (ThermoFisher Scientific, Cat. 4464058) using electroporation in T cell nucleofector media (Lonza). All cells were transfected with green fluorescente protein (GFP) plasmid and the fluorescence checked 24 hours before RNA extraction. Cells were incubated with gp91ds-tat (30µM) or sc-tat (30µM) for 24 hours and, after then, the RNA extracted using Trizol^{TM} Reagent (ThermoScientific, Cat.15596026) according to the manufacturer's instructions.

Superoxide production. Superoxide production was determined by flow cytometry using 10 μM dihydroethidium in $CD4^{+}CD25^{+}$ T cells purified from $Nox2^{fl/fl}FoxP3Cre^{+}$ and $Nox2^{fl/fl}$ mice, and stimulated with anti-CD3ε (4 μg/ml; BD Biociences, Wokingham, UK, Cat. 553238) plus anti-CD28 (8 μg/ml; Biolegend, San Diego, USA; Cat. 102102). Some cells were incubated with diphenyleneiodonium (DPI, 10μM) before TCR stimulation (negative control).

Cytokine production. Levels of IL-6, IL-10, CCL2 and IFN-γ were determined in heart homogenates and supernatant of T cell cultures using a Cytometric Bead Array kit (BD Pharmingen, Mouse Inflammation Kit, Cat. 552364). IFN-γ levels were also determined by ELISA (R&D, Cat. DY485-05). The cytokine levels were corrected by the total protein levels determined using Bradiford® reagent.

Quantitative PCR. RNA was extracted using Trizol™ Reagent (ThermoScientific, Cat.15596026) according to the manufacturer's instructions. After cDNA generation (GoScript™ reverse transcriptase, Promega, Cat. A5001), SYBR green real-time PCR was performed using the delta/delta Ct method and GAPDH for normalization. Primers were: NOX2 (forward, CCAACTGGGATAACGAGTTCAA; reverse, TCAGGGCCACACAGGAAAA), CCR4 (forward, ATCCTGAAGGACTTCAAGCTCCA; reverse, AGGTCTGTGCAAGATCGTTTCATGG), CCR8 (forward, GCAGTCTTTGAGGTGGTGGAAGC; reverse, TTGAATGGGACCCAGAAGAG), CXCR3 (forward, GTGGCTGCTGTGCTACTGAG; reverse, AAGGCCCCTGCATAGAAGTT), GAPDH (forward, CATCTTCTTGTGCAGTGC; reverse, CGGCCAAATCCGTTCAC), CCR1 (forward, ATTGGAGTGGTGGGCAATGTCCTA; reverse, AGCCAGGTTGAACAGGTAGATGCT), CCR3 (forward, TCCAGGTGTGGTGCTGTCGTTAAT; reverse, TTGGTATGTGAGGGCCGGTAGAAA), CCR5 (forward, ACTGCTGCCTAAACCCTGTCATCT; reverse, TTCATGTTCTCCTGTGGATCGGGT), CCR7 (forward, CCAGACCGTGGCCAATTTCAACAT; reverse,

ACAAGAAAGGGTTGACACAGCAGC), CCR9 (forward, CATCCATTGACCAGCAGCAGCAAT; reverse, CATCCATTGACCAGCAGCAGCAAT), CXCR5 (forward, AAGCGGAAACTAGAGCCTGGTTCA; reverse, ACCATCCCATCACAAGCATCGGTA), CXCR4 (forward, AGCTAAGGAGCATGACGGACAAGT; reverse AACGCTGCTGTAGAGGTTGACAGT), CXCR6 (forward, ACCTATGAGTGGGTCTTTGGCACA; reverse TGTGATGCAGGTGAGAGTGAGCAT), CXCR7 (forward, AGCTCATTGATGCCTCCAGAGTGT; reverse ATACCACTCAAGCAACCAGACCCA), CXCR1 (forward TGGCCTCCTTTCCTTTGTACACGA; reverse AAAGGAATTTCAAGCCCACCTGGC), CX3R1 (forward CGACATTGTGGCCTTTGGAACCAT; reverse AGATGTCAGTGATGCTCTTGGGCT); CCR6 (forward CCTCACATTCTTAGGACTGGAGC; reverse GGCAATCAGAGCT CTCGGA); CCR2 (forward GGCATTGGATTCACCACAT; reverse CAAGGCTCACCATCATCGTA); CXCR2 (forward CTCCCAAGCCTTGAGTCACA; reverse CCACCTTGAATTCTCCCATC); Human CCR4 (forward, CAGCTCCCTGGAAATCAACATTC; reverse TTGGCAGAGCACAAAAAGG), Human CCR8 (forward TCTGAAGATGGTGTTCTACA, reverse ACTTTTCACAGCTCTCCCTA), Human GAPDH (forward GTCTCCTCTGACTTCAACAGCG, reverse ACCACCCTGTTGCTGTAGCCAA).

PCR for miR-214. cDNA synthesis and q-PCR for miR-214-3p and miR-214-5p were done using miCury LNAtm MiRNA PCR Starter Kit, Mouse mmu-miR-214-3p and mmu-miR-214-5p (Qiagen, Product 339320) Cat. YKP-MM-YP00204510-YP00204575) according to the manufacturer's instructions.

EC purification and culture. ECs were purified according to a previously published protocol (5). EC medium consisted of Dulbecco's Modified Eagle media (DMEM, GIBCO, Cat. 41966-052) supplemented with 2 mM glutamine (GIBCO, Cat. 250-30), 50 IU/mL penicillin (GIBCO,

Cat. 15140-122), 50 mg/mL streptomycin (GIBCO, Cat. 15140-122), 50 mM 2-Mercaptoethanol (2-ME) (GIBCO, Cat. 31350-010), 1mM sodium pyruvate (GIBCO, Cat. 11360-039), 20mM N-2-hydroxyethylpiperazine-N'-2-ethane sulfonic acid (HEPES) (GIBCO, Cat. 15630-056), 1% non-essential amino acids (GIBCO, Cat. 11140-050), 20% FCS and 150 mg/ml EC growth supplement (Sigma-Aldrich, Cat. E0760). Medium was replaced every 48 hours. When confluent, cells were detached with trypsin/EDTA (GIBCO, Cat. T4049) and seeded in glass coverslips coated with fibronectin (10µg/ml).

In vitro adherence under flow. Flow experiments were performed using a 35mm diameter circular parallel flow chamber (Glycotech) and a shear stress of 1.5 dyn/cm^2 using a Harvard Apparatus 2000 PHD syringe pump. Perfusion media consisted of RPMI supplemented with L-glutamine 2mM, 10% FCS, penicillin/streptomycin, 50 µM β-mercaptoethanol, and 25 mM Hepes. Cardiac ECs were seeded onto glass coverslips 35 mm in diameter (no. 1 thickness; VWR) precoated with 10µg/ml fibronectin. Before each perfusion assay, ECs were stimulated for 18 hours with 10 ng/ml recombinant murine TNF-α (PrepoTech, Cat. 315-01A). Each perfusion assay was performed during 30 minutes and Tregs were perfused at a density of 0.5x10^6 WT Tregs plus 0.5x10^6 Nox2-deficient Tregs per millilitre. After perfusion, 5 random fields were pictured and evaluated regarding the number of adhered Tregs. The experiments were repeated four times and the total number of leukocytes adhered to ECs in all fields are reported.

Adoptive transfer of purified Tregs. Tregs from WT or Nox2-deficient mice were purified from lymph nodes and spleens. 1 x 10^6 WT Tregs were stained in green (CellTracker™ Green CMFDA Dye; Cat.C7025) and 1 x 10^6 Nox2-deficient Tregs were stained in orange (CellTracker™ Orange CMFDA Dye; Cat.C2927) as previously described (6). Cells were mixed 1:1 and maintained overnight in RPMI media supplemented with 10% fetal calf serum, glutamine 2mM and penicillin-streptomycin (100IU and 0.1 mg/ml). Cells were cultured in the

presence of 2 x 10^5 irradiated dendritic cells (DCs) in a 24-well plate. DCs were purified by negative selection from bone marrow using FlowComp Dynabeads (Cat. 11463D), anti-CD4 clone YTS191.1, anti-CD8 clone YTS169.4, anti-B220 clone RA3-6B2. DCs were grown in GM-CSF 20 mg/ml for 7 days and were then irradiated at 30Gy for 10 minutes.

Immediately after the heart transplant surgery, cells were washed in PBS and injected into the inferior vena cava above the local anastomosis. Heart allografts were harvested 5 days after the surgery and the stained cells evaluated by flow cytometry.

Actin polymerization. Purified Tregs were stimulated for one minute with CCL1 (250ng/ml) or CCL22 (100ng/ml) and fixed in 4% paraformaldehyde (PFA). Cells were then permeabilized with 0.2% Triton-X and stained with Phalloidin-tetramethylrhodamine B isothiocyanate (Sigma; Cat.P1951) for 2 hours. Images were acquired in a confocal TCS SP5 (Leica, London UK) microscope (63X). The mean fluorescence intensity (MFI) was determined in 40 cells from five randomly chosen fields using Image J software (Imaging process and analysis in Java, National Institute of Mental Health, USA).

Chemotaxis assay. Chemotaxis was assayed in a 24-transwell chamber using a 5-µm-pore insert (Sarstedt, Cat. 833932500). Tregs (2×10^5 cells) were allowed to migrate toward CCL22 (250 ng/ml) or CCL1 (100ng/ml) in RPMI culture medium at 37°C and 5% CO_2. After 6 hours, the up insert was removed and the cells on the bottom of the chamber counted using a Neubauer Haemocytometer.

ICAM-1 activation and binding. Soluble FITC ICAM-1-Fc-F(ab')$_2$ complexes were generated by incubating FITC-labeled goat anti-human IgG F(ab')$_2$ fragments (109-135-098, Jackson Immunoresearch; 1:6.25) with ICAM1-Fc (200µg/ml) in HBSS for 30minutes at 4° C. Purified $CD4^+CD25^+$ Tregs pre-stained with anti-CD4 PeCy7 (BD Biosciences; Cat. 552775) were

incubated with FITC ICAM-1-Fc-F(ab')$_2$ complexes and stimulated with CCL22 (250 ng/ml) up to 5 minutes. Cells were fixed in 4% PFA and evaluated by flow cytometry.

Luciferase reporter assays. The 3' untranslated region (UTR) of mouse CCR4 and part of the coding region (CD) harbouring putative binding sites of the miR-214-3p as predicted by miRWalk 2.0 and TargetScan 7.2 were cloned into the dual-luciferase reporter vector psiCheck2 (Promega). The following primer sets were used: mCCR4 UTR (forward 'ATATCTCGAGTGGCGTTAACAAGCTCCACA'; reverse 'CGACCGTTTAAACGTCAGATCCTCATTTCTCC'); mCCR4 CD (forward 'ATATACTCGAGGCTCAACTGTTCTCATTGGC'; reverse 'GTGTGGTTTAAACAAAAACCCACTGGTCGG'). Part of the coding region of mouse CCR8 harbouring putative binding sites of the miR-214-3p as predicted by miRWalk 2.0, was cloned into the dual-luciferase reporter vector psiCheck2 (Promega). The following primer sets were used: mCCR8 CD (forward 'ATATCTCGAGTGGCCATCTTGTACTGCGTC'; reverse 'CGACGTTTAAACCATTGTAGCATGCCGTCTTC'). Jurkat cells (clone EC6-1, ATCC TIB-152) were cultured with Human T activator CD3/CD28 expansion beads (1:2, Miltenyi Biotec, Cat. 130-095-345) for 3 hours. Then, the cells were transfected with 5µg of the luciferase constructs using electroporation in T cell nucleofector media (Lonza, Cat. VPA-1002). Luciferase activities in cell lysates were determined using the Dual-Glo luciferase system (Promega) in a plate luminometer (Mithras LB 940, Berthold)

Statistics. Analyses were performed using GraphPad Prism software v5.0 (GraphPad Software, Inc., La Jolla, CA). Data are reported as mean ± SEM. Comparisons were undertaken using Kruskal-Wallis followed by Dunn's post-test or Mann Whitney t-test or 2-way ANOVA followed by Bonferroni post-test, as appropriate. $P<0.05$ was considered significant.

References

1. Hasegawa T, Visovatti SH, Hyman MC, Hayasaki T, and Pinsky DJ. Heterotopic vascularized murine cardiac transplantation to study graft arteriopathy. *Nat Protoc.* 2007;2(3):471-80.
2. Sag CM, Schnelle M, Zhang J, Murdoch CE, Kossmann S, Protti A, et al. Distinct Regulatory Effects of Myeloid Cell and Endothelial Cell Nox2 on Blood Pressure. *Circulation.* 2017.
3. Zhou X, Jeker LT, Fife BT, Zhu S, Anderson MS, McManus MT, et al. Selective miRNA disruption in T reg cells leads to uncontrolled autoimmunity. *The Journal of experimental medicine.* 2008;205(9):1983-91.
4. Pollock JD, Williams DA, Gifford MA, Li LL, Du X, Fisherman J, et al. Mouse model of X-linked chronic granulomatous disease, an inherited defect in phagocyte superoxide production. *Nat Genet.* 1995;9(2):202-9.
5. Marelli-Berg FM, Peek E, Lidington EA, Stauss HJ, and Lechler RI. Isolation of endothelial cells from murine tissue. *J Immunol Methods.* 2000;244(1-2):205-15.
6. Quah BJ, Warren HS, and Parish CR. Monitoring lymphocyte proliferation in vitro and in vivo with the intracellular fluorescent dye carboxyfluorescein diacetate succinimidyl ester. *Nat Protoc.* 2007;2(9):2049-56.

Supplementary Figures

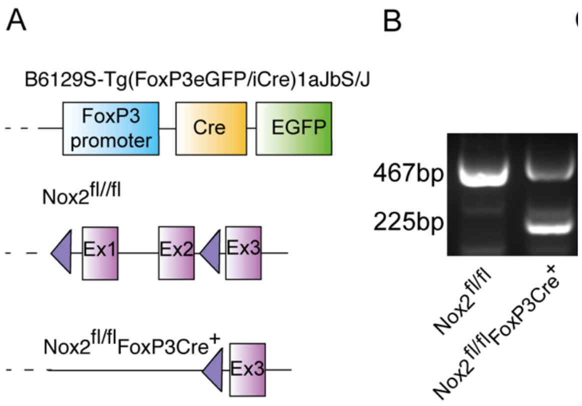

Supplementary Figure 1. Generation of Nox2$^{fl/fl}$FoxP3Cre^{+} mice. (A) Schematic representing the generation of Nox2$^{fl/fl}$FoxP3Cre^{+} mice. (B) PCR showing the DNA fragment indicative of Nox2 recombination in Nox2$^{fl/fl}$FoxP3Cre^{+} mice lymph nodes.

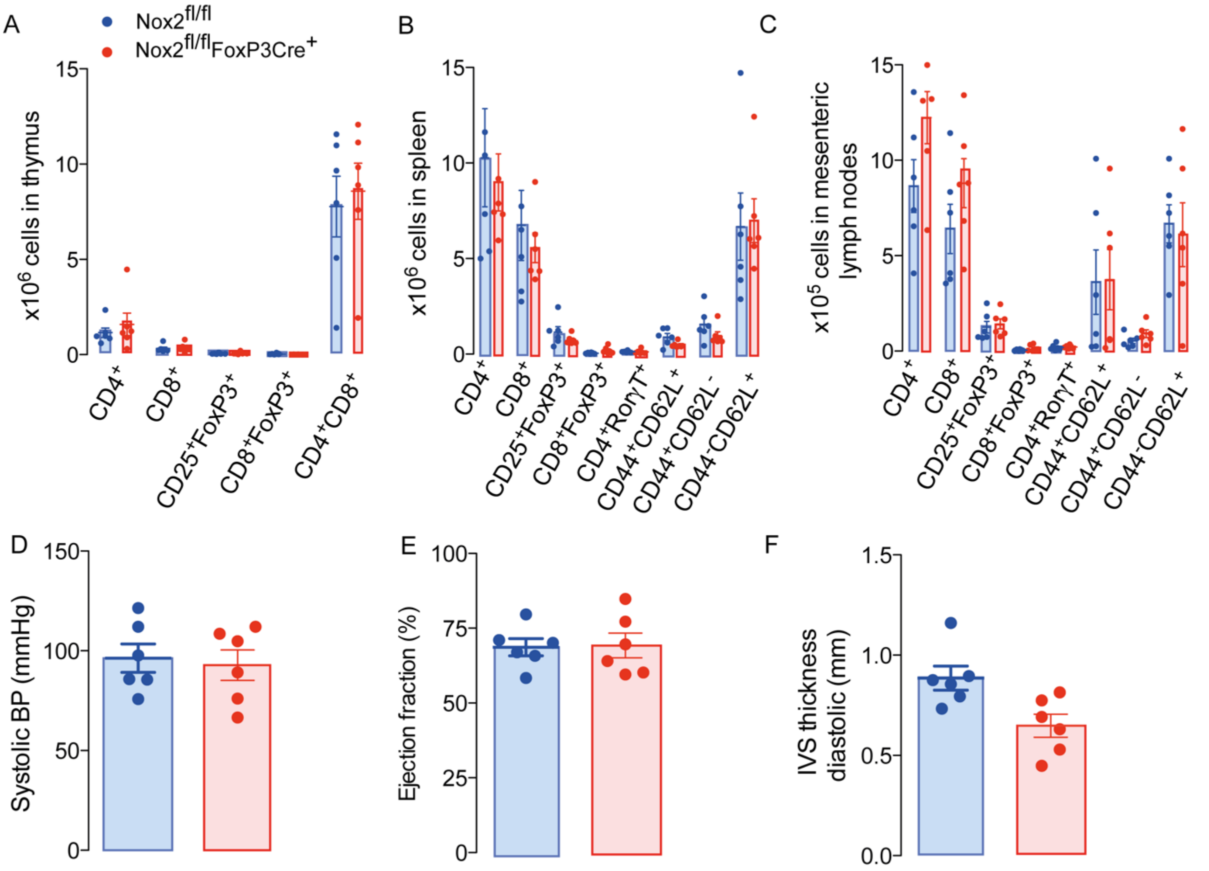

Supplementary Figure 2. Nox2$^{fl/fl}$FoxP3Cre^{+} mice have preserved cardiovascular parameters and distribution of T cells in lymphoid tissue under baseline conditions. (A-C) Absolute numbers of different populations of CD4^{+} and CD8^{+} cells in thymus (A), spleen (B) and mesenteric lymph nodes (C) by multi-colour flow cytometry. (D) Systolic blood pressure (BP) measurements by tail-cuff plethysmography, (E) Left ventricular ejection fraction and (F) diastolic interventricular septum thickness (IVS) by echocardiography in 8-10 week old mice (n=6 per group).

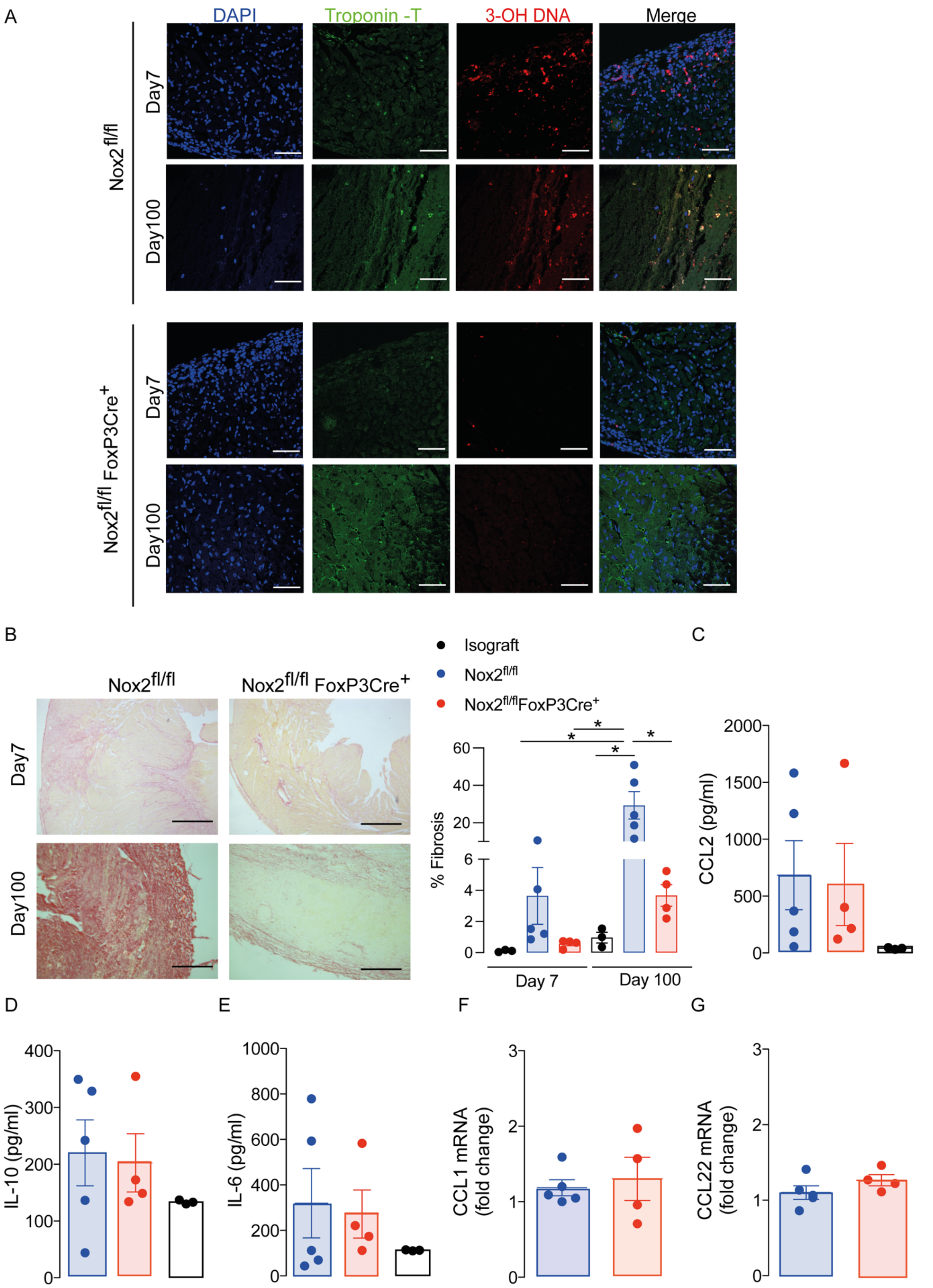

Supplementary Figure 3. Allografts transplanted in Nox2$^{fl/fl}$FoxP3Cre^{+} mice have lower cardiomyocyte necrosis and fibrosis. Mice with FoxP3-targeted Nox2 deletion

(Nox2$^{fl/fl}$FoxP3Cre^{+}) and littermate controls (Nox2$^{fl/fl}$) were transplanted with heart allografts from CB6F1 mice. Mice transplanted with hearts from B6 mice were used as isograft controls. (A) Representative images showing necrosis (red) of cardiomyocytes stained with troponin-T (green) in allografts 7 and 100 days after surgery. Nuclei were stained with DAPI (blue). Scale bar: 50µm. (B) Percentage of fibrosis in allografts by picrosirius red staining (PRS) 7 and 100 days after surgery. Representative images in bright-field are shown on the left and mean data on the right. Scale bar: 25µm. (C-E) Cytokine levels in heart allograft homogenates. (F-G) CCL22 and CCL1 mRNA levels in heart allografts, 7 days after surgery. Graphs represent mean ±SEM (n=4-5 per group); *P<0.05 for indicated comparisons. Kruskal-Wallis followed by Dunn's post test in B.

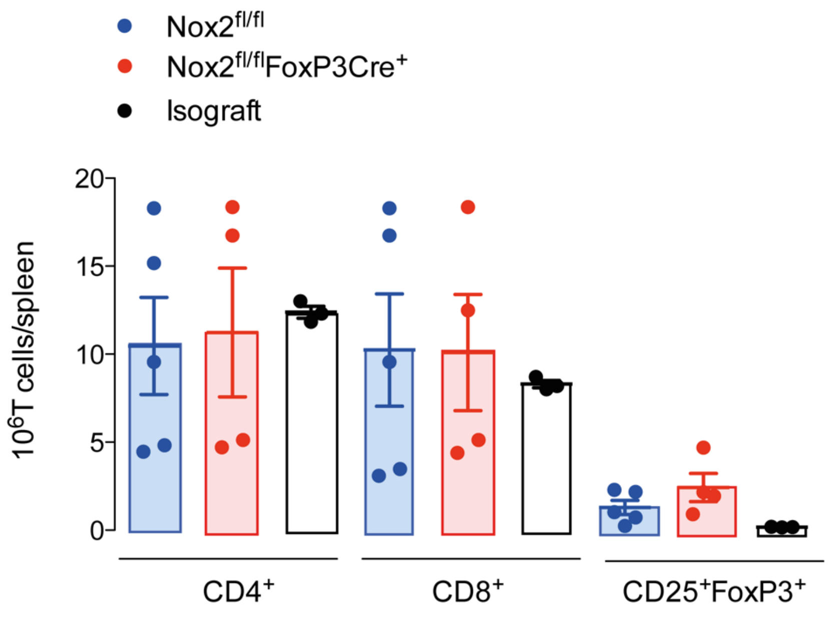

Supplementary Figure 4. Splenic leukocytes are not affected by Treg-targeted Nox2 deletion. Mice with FoxP3-targeted Nox2 deletion (Nox2$^{fl/fl}$FoxP3Cre^{+}) and littermate controls (Nox2$^{fl/fl}$) were transplanted with hearts from CB6F1 mice. Hearts from B6 donors transplanted into *Nox2*$^{fl/fl}$ recipients were used as isograft controls. CD4^{+}, CD8^{+} and CD25^{+}FoxP3^{+} cells in spleen 7 days after surgery. Graph represents mean ±SEM (n=4-5 per group).

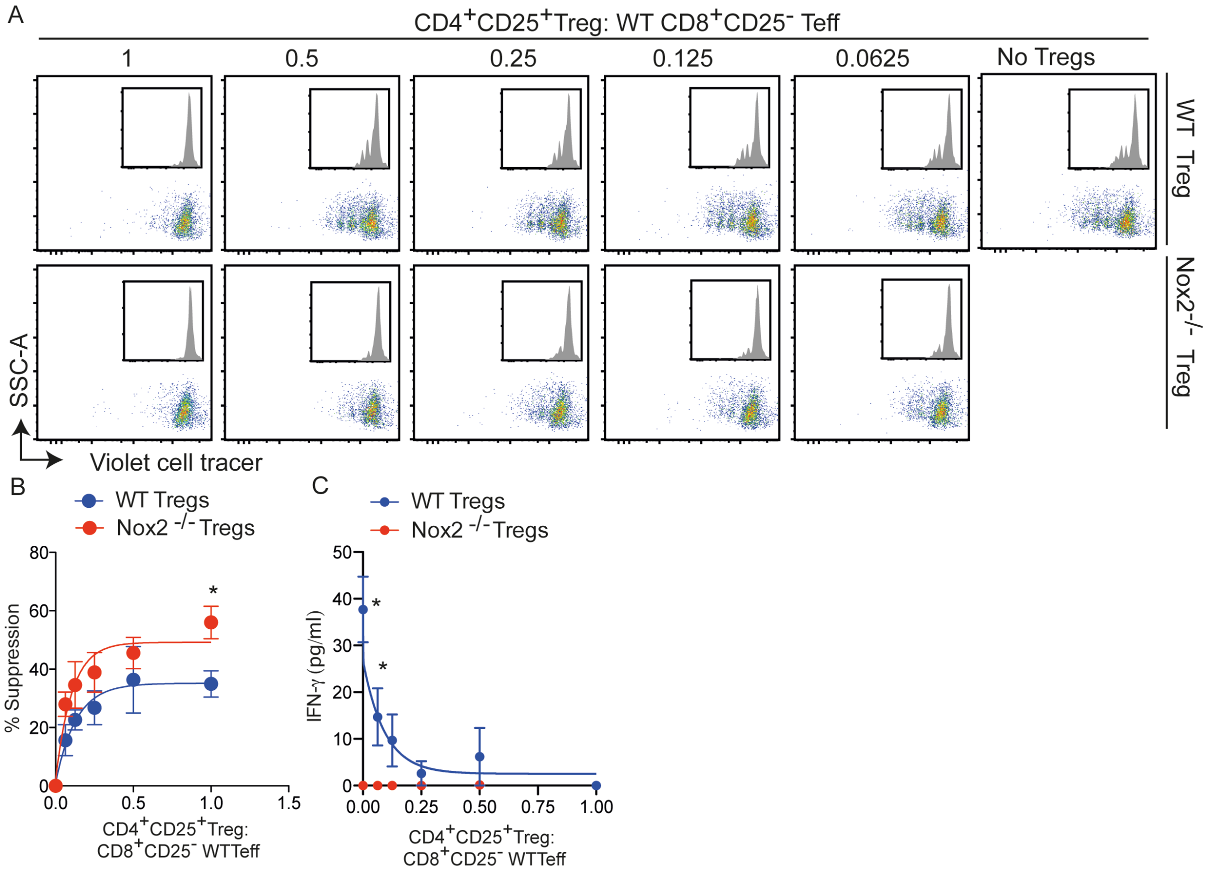

Supplementary Figure 5. Tregs deficient of Nox2 have higher suppressive activity against CD8$^+$CD25$^-$ cells than do WT Tregs. (A, B) In vitro suppression assay using CD4$^+$CD25$^+$ Tregs purified from spleen and lymph nodes of Nox2-deficient mice (Nox2$^{-/-}$) and WT littermate controls. Cells were stimulated with APCs and anti-CD3ε antibody. Representative plots in A show proliferation of CD8$^+$CD25$^-$ Teffs after 3 days of stimulation; numbers at the top are the ratio of CD4$^+$CD25$^+$ Tregs to Teffs. Mean data is shown in B. (C) Levels of IFN-γ in supernatant of cultures. *$P<0.05$. 2-way ANOVA followed by Bonferroni's post-test. Data represents one of two experiments realized in triplet.

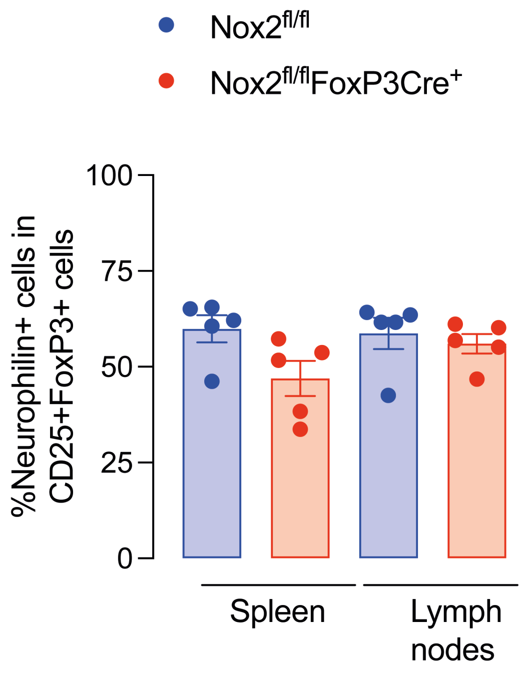

Supplementary Figure 6. Tregs from spleen and lymph nodes of Nox2$^{fl/fl}$FoxP3Cre^{+} and littermate controls Nox2$^{fl/fl}$ are neurophilin-1^{+}. Percentages of neurophilin-1^{+} cells in CD4^{+}CD25^{+}FoxP3^{+} cell population (n=5 per group).

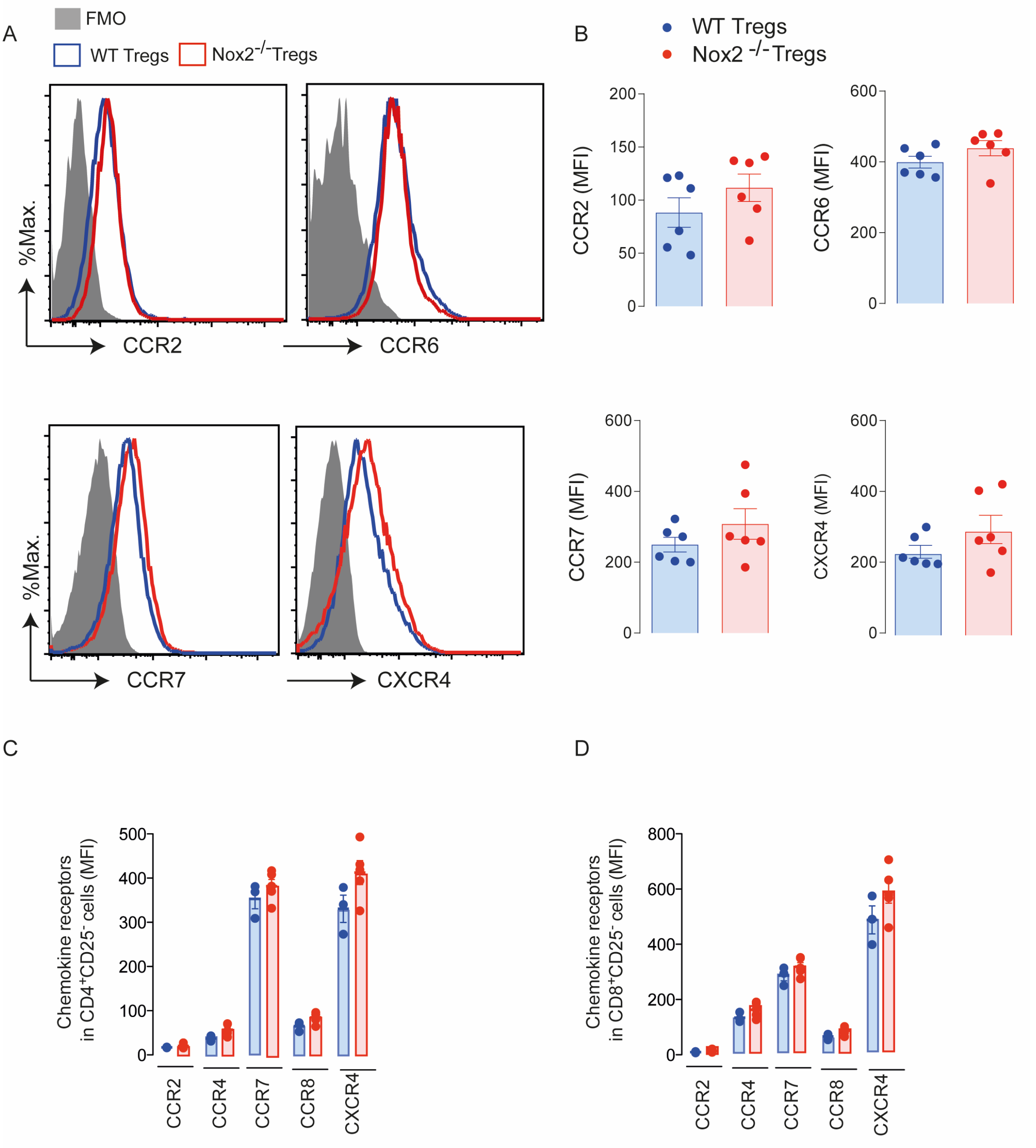

Supplementary Figure 7. Nox2-deficient CD4$^+$CD25$^+$ Tregs have comparable protein levels of CCR2, CCR6, CCR7 and CXCR4 to WT Tregs. (A-B) Histograms (A) and bar graphs (B) representing the chemokine receptor protein levels by flow cytometry in CD4$^+$CD25$^+$ cells (n=6 per group). (C-D) Protein levels of chemokine receptors in CD4$^+$CD25$^-$ (C) and CD8$^+$CD25$^-$ cells (D). MFI: median fluorescence intensity (n=3-4 per group).

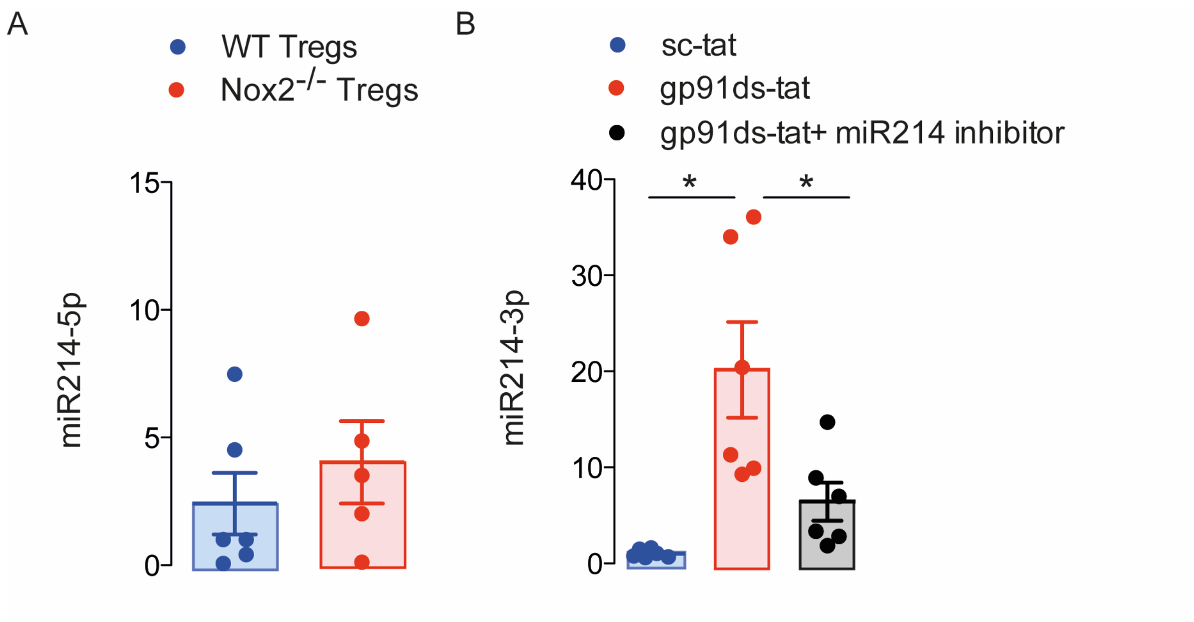

Supplementary Figure 8. miR-214-5p is not affected whereas miR-214-3p is enhanced by Nox2 deletion or inhibition in Tregs. (A) Expression of miR-214-5p in Nox2-defcient or WT CD4$^+$CD25$^+$ Tregs. (B) Expression of miR-214-3p in Jurkat T cells transfected with miR-214-3p inhibitor and incubated with gp91ds-tat (30μM). Graphs represent mean ±SEM (n=5-6 per group); *P<0.05 for indicated comparisons; Kruskal-Wallis followed by Dunn's post-test.

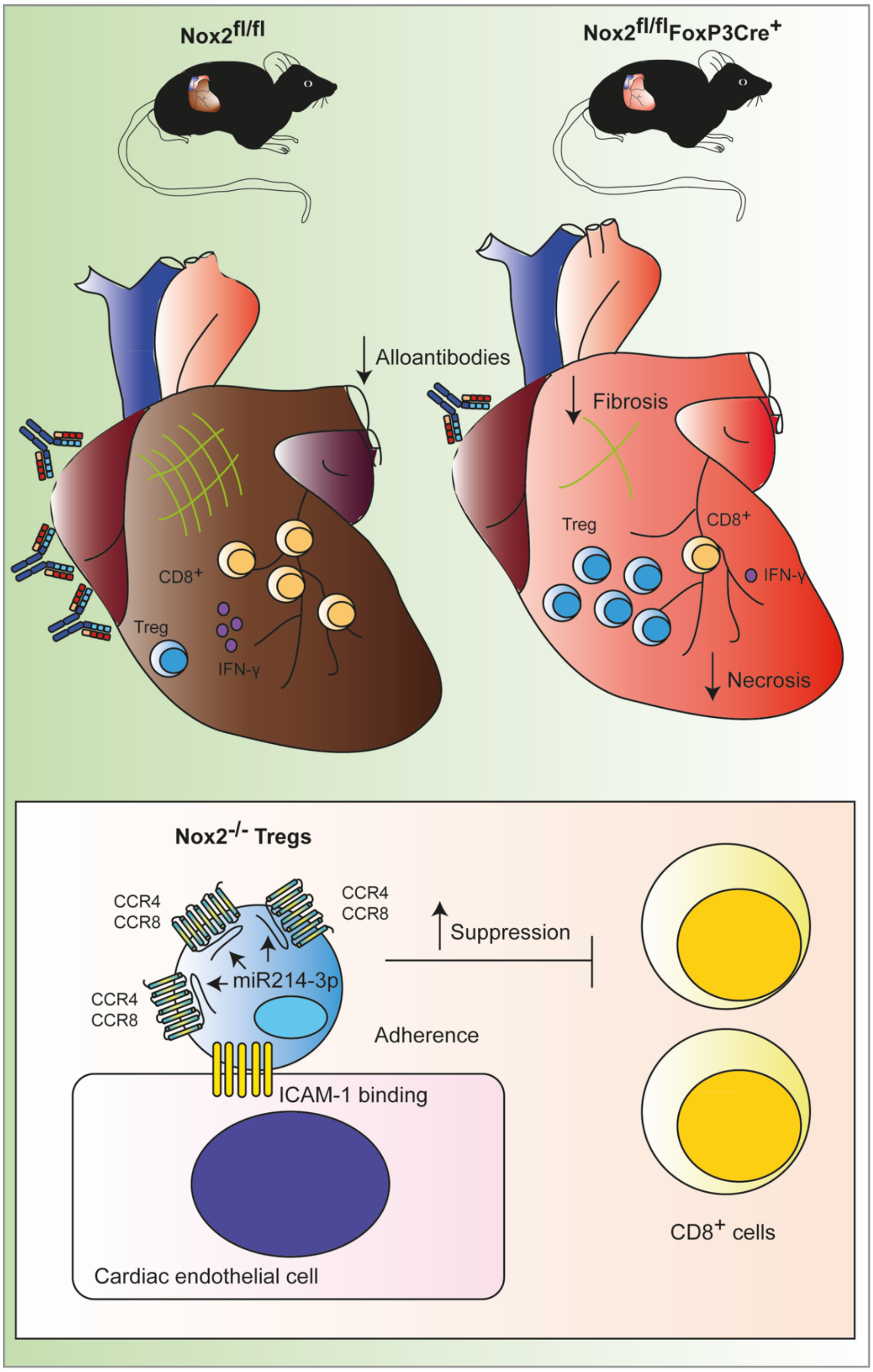

Supplementary Figure 9. Schematic of the mechanism through which Nox2-deficient Tregs improve cardiac transplantation outcome. Nox2-deficient Tregs express higher levels of miR214-3p, CCR4 and CCR8 chemokine receptors driving higher ICAM-1 activation and adherence to cardiac ECs (lower panel). The higher Treg chemotaxis into heart contributes to lower proliferation of $CD8^{+}$ T cells, IFN-γ production, and, as a result, the allograft shows reduced necrosis and fibrosis (up panel).

Supplementary video 1. Echocardiography video (long axis) of CB6F1 heart allograft transplanted into Nox2$^{fl/fl}$ control mice 10 days after surgery.

Supplementary video 2. Echocardiography video (long axis) of CB6F1 heart allograft transplanted into Nox2$^{fl/fl}$FoxP3Cre^{+} mice 16 days after surgery.
